# Editorial: Next-generation sequencing in ophthalmology: The microbiome in ocular health and disease

**DOI:** 10.3389/fcimb.2022.1080932

**Published:** 2022-11-10

**Authors:** Hiroshi Eguchi, Jerome Ozkan, Martin J. Holland

**Affiliations:** ^1^ Department of Ophthalmology, Faculty of Medicine Kindai University, Osaka, Japan; ^2^ School of Optometry and Vision Science, School of Biological, Earth and Environmental Sciences, University of New South Wales, Sydney, NSW, Australia; ^3^ London School of Hygiene and Tropical Medicine, University of London, London, United Kingdom

**Keywords:** next-generation sequencing, ocular surface microbiome, gut microbiome, brain-gut axis, 16S rRNA gene sequencing

Application of next-generation sequencing (NGS) technology in infectious diseases has allowed the simultaneous analysis of genome information from a myriad of microorganisms. This is known as meta-genomics. NGS technology can facilitate rapid and agnostic characterization of clinical specimens in a relatively short time and at a low cost enabling it to be utilized in many clinical settings. Most studies analyze organs where the number of colonizing bacteria is large e.g., the gut and oral cavity. As a result, the gut microbiome reportedly influences diseases in these organs, but it can also affect disorders in organs outside the digestive tract, such as neurological disorders [e.g., Parkinson’s disease, Alzheimer’s disease and multiple sclerosis (Dinan and Dinan, 2022)] in addition to the success of therapy for several types of cancer (Lu et al., 2022).

NGS microbiome studies now encompass several organs and anatomical sites with relatively low microorganism biomasses that were often considered “sterile”, such as the ocular surface. Working with these sites and specimens requires continuous caution when interpreting analyses, since there remains skepticism around viability and the contribution of the local microbial ecosystem. Differences in the specimen collection methods and research protocols, as well as contamination during experiments, have the potential to disproportionately affect results in low biomass sites and samples, thus greatly influencing these results. Consequently, reports on the normal ocular surface microbiome (OSM) continue to generate mixed results (Dong et al., 2011; Zhou et al., 2014; Shin et al., 2016; Eguchi et al., 2017; Ozkan et al., 2017; Zilliox et al., 2020; Matysiak et al., 2021). Although a rigorous understanding of the true ocular surface microbiome is desirable, the direction of its research is gradually changing, with many comparative studies between the OSM of normal subjects and those with ocular surface disease. Each of these published studies utilize methods developed at each institution which would benefit from harmonization in the future. Over time, we expect these studies may uncover new insights into the pathophysiology of ocular surface diseases and development of new treatment methods.

Reports included in this Research Topic consider the OSM or the gut microbiome related to pathogenesis of the ocular surface disease. They investigate the OSM and bacterial keratitis (Ren et al.), dry eye (Qi et al.), cicatricial keratoconjunctivitis (Ueta et al.), and blepharitis (Fu et al.). Analysis of the OSMs in these cases provides molecular biological insights into the pathogenesis of opportunistic infections. Diagnosis of infectious keratitis and annotation of microbiome information for such cases through artificial intelligence (AI) machine learning may lead to the widespread use of precision medicine for bacterial keratitis (Ren et al.). For dry eye and cicatricial keratoconjunctivitis, quality and/or quantity of tear fluid, is altered, while eyelid findings affect the ocular surface in blepharitis. Therefore, it is not difficult to imagine that these conditions may affect the OSM in these diseases. However, as eye drops and oral medications are often administered simultaneously, verification of the association between these medications and the gut microbiome is necessary. With respect to the influence of the gut microbiome on ocular disease the reports by Xue et al., and Hou et al., Low et al. seemingly suggest that changes in the gut microbiome are not a direct factor in ocular manifestations, but instead may exhibit an indirect effect. However, the possibility of managing the underlying disease using probiotics has been postulated, and this exciting area of research is expected to lead to the rapid development of future treatments.

Limitations in current NGS research in ophthalmology includes paucity of metagenomic analysis and reports on virome analysis. It has long been known that HSV-1 shedding is present in the tear fluid for herpes keratitis, and results of its quantitative analysis using real-time PCR have been reported in relation to disease status (Kakimura-Hasegawa et al., 2008). However, there may be pathological conditions that cannot be diagnosed by only understanding the viral load using molecular biological methods targeting specific viruses. Presumably, the ocular surface, which is externally exposed, may be consequently exposed to viruses from various animal and plant origins. If a bacterial flora inhabits the ocular surface, a phageome will also exist. Ocular surface virome analysis is considered an urgent issue in the discovery of idiopathic viral ocular infections and for more detailed understanding of the pathogenesis of the ocular surface bacterial infections. Additionally, it is still unclear in many cases whether microbiome dysbiosis in clinical specimens represents the cause or result of a disease. Future studies correlating patient symptoms, findings, course, and medication status with their microbiome may resolve this problem. However, animal experiments using disease models and *in vitro* experiments that scrutinize the relationship between microbiome alteration and host responses are still necessary.

In conclusion, the application of NGS technologies in ophthalmology is far from routine but its increasing use has the potential to contribute to the elucidation of pathogenesis, development of new treatment methods and response to treatment, not only for ocular surface diseases, but also for ocular diseases where there is interaction between host microbiome(s) and their metabolites.

## Author contributions

All authors listed have made a substantial, direct, and intellectual contribution to the work and approved it for publication.

## Conflict of interest

Author HE received financial support from Novartis Pharma and Alcon Pharma. The authors declare that the research was conducted in the absence of any commercial or financial relationships that could be construed as a potential conflict of interest.

## Publisher’s note

All claims expressed in this article are solely those of the authors and do not necessarily represent those of their affiliated organizations, or those of the publisher, the editors and the reviewers. Any product that may be evaluated in this article, or claim that may be made by its manufacturer, is not guaranteed or endorsed by the publisher.
